# Prognostic factors and related complications/sequalae of squamous cell carcinoma located in the gingivobuccal complex

**DOI:** 10.1186/s12957-022-02708-w

**Published:** 2022-07-26

**Authors:** Yunhao Zhu, Bo Li, Huan Liu, Delong Li, Aoming Cheng, Chong Wang, Zhengxue Han, Zhien Feng

**Affiliations:** Department of Oral and Maxillofacial-Head and Neck Oncology, Beijing Stomatological Hospital, Capital Medical University, No. 4; Tian Tan Xi Li, Dongcheng District, Beijing, 100050 P.R. China

**Keywords:** Gingivobuccal complex cancer, Oral squamous cell carcinoma, Bone invasion, Hardware-related complication, Sequalae

## Abstract

**Background:**

Gingivobuccal complex (GBC) was a relatively new concept of oral subsite that was comprises of the upper and/or lower gingiva, gingival buccal sulcus, and adjacent buccal mucosa. Squamous cell carcinoma (SCC) of the GBC had a poor prognosis, with few studies analyzing this particular entity. The objective of this study was to analyze the risk factors affecting the prognosis and complications/sequalae of gingivobuccal complex cancer.

**Methods:**

Between December 2014 and August 2019, a total of 122 patients diagnosed with primary gingivobuccal complex cancer in Beijing Stomatological Hospital, Capital Medical University were enrolled in the study. Through outpatient reviewed and telephone followed-up for 2-5 years postoperatively, postoperative relapse and complications/sequalae were assessed. The primary outcome parameter was 2-year disease-free survival.

**Results:**

The most common central site of the tumor was the buccal mucosa (45.1%), followed by the lower gingiva (36.9%). The most diseases were pT4a (45.1%) and there was lymph node invasion (pN+) in 41.8% of patients. Moderate differentiated squamous carcinoma (77.9%) accounted for the vast majority of the histopathological differentiation. A total of 62.3% of tumors invaded the bone, while, 5.7% invaded the skin layer. Survival analysis found that 44.3% of patients experienced relapse within two years postoperatively and the mortality rate after relapse was 75.9%. Almost 60.0% of the tumors involving the maxilla and/or mandible developed relapse. Cox proportional hazards model found that pN stage (p= 0.002) and bone invasion (p= 0.007) were significant independent predictors of 2-year disease-free survival. Importantly, 63.1% of patients had postoperative (and postradiotherapy) complications/sequalae. It was noteworthy that 18 of 43 patients (41.9%) who implanted with titanium plates had hardware-related complications/sequalae, and the most of them were titanium plate exposure (61.1%).

**Conclusions:**

Squamous cell carcinoma of the gingivobuccal complex cancer, as a new subsite worthy of attention in oral cancer, has a high complication/sequalae rate, high relapse rate and poor prognosis.

**Trial registration:**

Prospective, Observational, Real-world Oral Malignant Tumors Study (clinicaltrials.gov identifier: NCT02395367). The approval of the Institutional Review Board of the Beijing Stomatological Hospital of Capital Medical University (Approval number: CMUSH-IRB-KJPJ-2015-08)

## Background

According to Global Cancer Statistics 2018, the incidence of lip cancer and oral cancer ranked 18th among all cancers [1]. Since the oral cavity is located at the beginning of the digestive tract, it bears huge physiological and aesthetic functions, and oral cancer has a great impact on the survival and prognosis of patients. Tobacco and alcohol consumption were the major independent risk factors of oral squamous cell carcinoma (OSCC) [2, 3]. Squamous cell carcinoma (SCC) of the gingivobuccal complex (GBC) is a relatively new concept of oral cancer subsite that comprises the upper and lower gingiva, gingival buccal sulcus, adjacent buccal mucosa and retromolar triangle. It is uncommon in Western countries, accounting for only 10% of oral cancers [4]. However, it accounts for 40% of oral cancers in Southeast Asia, South-central China and Africa [5, 6]. There were even reports in the literature that its incidence in India was as high as 72% [7]. The habit of chewing betel nuts and shredding tobacco is the main reason for the high risk of developing SCC of GBC. Compared with other common oral cancers, such as tongue and floor of mouth cancer, SCC of GBC invades more anatomical structures. It more easily invades the mandible and skin and spreads to cervical lymphatic tissue due to its close proximity [5, 7–9].

The current treatment of oral SCC is surgical resection with or without adjuvant radiotherapy/chemoradiotherapy. Most patients require radical resection, including a large amount of soft tissue around the GBC coupled with partial or segmental resection of the jaw; therefore, reconstruction of the complex tissue defect is necessary. Titanium plates are often used to provide rigid internal fixation after bone defect repair and reconstruction [10]. However, oral cavity cancers simultaneously involved in the gingiva and buccal area still have very high locoregional failure rates (35%) [7]. The factors leading to a worse prognosis have not been fully studied. Unfortunately, since patients with GBC cancer usually present at a more advanced stage, once they undergo postoperative radiotherapy, more complications will occur [11, 12]. These complications seriously affect patient prognosis and cause a large burden on life.

More focus on this independent cancer subsite will provide a good foundation for improving treatment, reducing complications/sequalae and enhancing the prognosis of GBC cancers. The objective of this study was to analyze the risk factors affecting the prognosis and complications/sequalae of GBC cancers.

## Patients and methods

### Patients

This prospective cohort study was conducted in accordance with established ethical principles, including the World Medical Association Declaration of Helsinki (2002 version), and with the approval of the Institutional Review Board of the Beijing Stomatological Hospital of Capital Medical University (Approval number: CMUSH-IRB-KJPJ-2015-08). The data of this study originated from POROMS and the pre-experiment of this clinical trial, a Prospective, Observational, Real-world Oral Malignant Tumors Study (clinicaltrials.gov identifier: NCT02395367). All patients were treated at the Department of Oral and Maxillofacial-Head and Neck Oncology, Beijing Stomatological Hospital, Capital Medical University and were pathologically diagnosed with OSCC between December 2014 and August 2019. The inclusion criteria were as follows: (1) primary tumor located in the GBC, (2) pathological diagnosis of SCC, (3) pathology reports negative margin status, and (4) no evidence of distant metastasis. The exclusion criterion was patients who were found to have unresectable disease at the time of surgery. There were 122 patients who fulfilled the inclusion criteria and were included in this study.

All study participants were obtained preoperative chest X-ray, complete blood counts, and blood chemistries; CT, MRI, and/or positron emission tomography (PET) scans were used preoperatively to determine the extent of the tumor. The TNM stage of the tumor was determined based on clinical and imaging findings according to the American Joint Committee on Cancer (AJCC), 8th edition staging criteria.

### Treatment

According to the American Joint Committee on Cancer (AJCC) 2017 8th edition TNM staging and the National Comprehensive Cancer Network (NCCN) guidelines for diagnosis and treatment of oral cancer, only primary tumor resection was performed for patients with cT1-2, N0. For the remaining patients, primary tumor resection was accompanied by lymph node dissection. Adjuvant radiotherapy was advised for pathology returning positive lymph nodes. Adjuvant radiotherapy was scheduled within 4-8 weeks after surgery. The prescribed dose was 1.8-2Gy per fraction per day, 5 days per week, for a total dose of 60 Gy. The total radiation dose for patients with multiple positive cervical lymph nodes was 66 Gy. Concomitant chemoradiotherapy with cisplatin at 30 mg/m2 per week was recommended for patients with pathologically proven positive margin and/or positive extracapsular invasion [13].

**Fig. 1:**
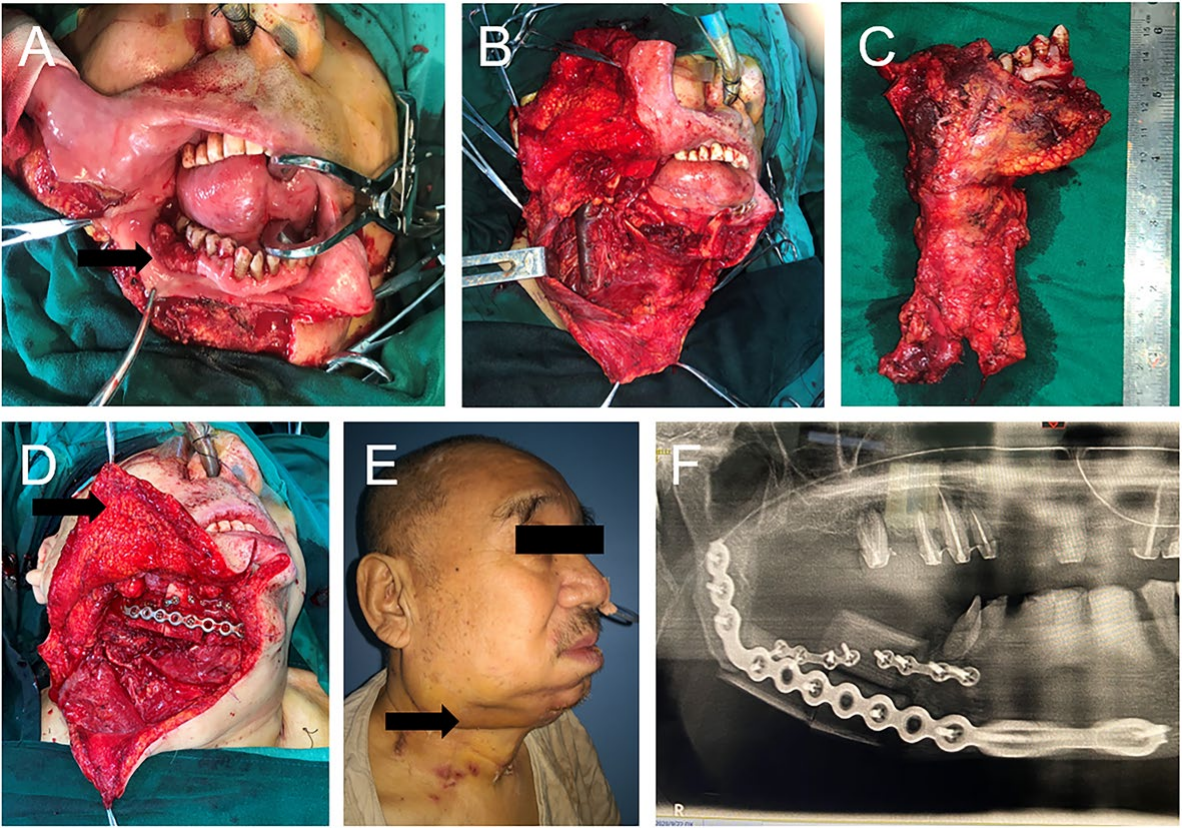
Typical primary tumor of GBC cancer (**A**); The defect after en bloc resection of the primary tumor and neck lymph tissue (**B**); Specimens of primary tumor and neck dissection (**C**); Reconstruction using a free fibula flap and rigid internal fixation (the arrow shows that only the skin and subcutaneous tissue will cover the surface of the fibular flap and the reconstructed titanium plate, and the platysma and buccal muscles have been removed) (**D**); Profile image at one week postoperatively (the arrow shows that only the thin layer of skin and subcutaneous tissue was wrapped on the outside of the titanium plate) (**E**); Orthopantomography one week postoperatively (**F**)

The operating procedure and short-term postoperative results of a typical GBC cancer case are shown in Fig. 1. In brief, the level of tissue resection was determined according to the depth of invasion of the primary tumor. At least the buccal muscle and platysma were not preserved. After the tumor was exposed, the primary tumor and cervical lymph node were resected en bloc. After resection of the mandibular segment, the soft and hard tissue defects were repaired with free flaps. The biggest feature of GBC cancer is that thin skin directly covers the surface of the bone flap or hardware.

### Follow-up strategy

Patients were advised to review every 3 months for 2 years after surgery and every 6 months for the third, fourth and fifth years. For patients who were unable to be reviewed, the follow-up was changed to telephone. Ultrasound was performed every 3 months and CT/MRI every 6 months. The above policy for follow-up has been a routine practice in our hospital.

### Predictors and outcome variables

Predictive variables included demographics (age and sex), anatomy (T stage, N stage, depth of invasion and growth pattern), lifestyle habits (tobacco, betel nuts and alcohol use), tumor involvement (skin, maxilla or mandible), etc. The main outcome assessment parameter was 2-year disease-free survival (DFS). DFS was calculated as the time from the first operation to the time of the first recurrence in the primary site, cervical lymph nodes, distant metastasis, second primary malignancy or death.

### Data analysis

The cutoff date for all patients was August 2021. Descriptive statistics are expressed as frequencies, percentages, and means ± standard deviations as appropriate. The baseline demographic data were compared using the chi-square test for categorical variables. The Kaplan-Meier method was used to derive estimates of the 2-year DFS. Statistical significance was determined using the log-rank test. A Cox proportional hazards model was used to adjust for the effects of potential confounders. Factors that were statistically significant in the univariate analysis of the Cox proportional hazards model were included in the multivariate analysis using the “Forward Method”. All tests were two-sided, and p values less than 0.05 were considered statistically significant. All statistical analyses were performed using SPSS software, version 21.0 for Windows (SPSS Inc., Chicago, IL, USA).

## Results

### Patient factors

In total, 122 patients were enrolled in this study, including 67 (54.9%) male and 55 (45.1%) female. The mean age was 63 years (range: 31-85). Fifty-one (41.8%) patients had a habit of smoking, and 37 (30.3%) patients had a habit of drinking. Because our institution is located in northern China, only 2 (1.6%) patients had a habit of chewing betel nuts. The median time interval between the onset of symptoms and the hospital visit was 1.0 months (average: 5.5 months; range: 0-240 months). The baseline demographic and clinical characteristics are summarized in Table 1.

**Table 1:** Baseline demographic and clinicopathological characteristics

Category	Number	Proportion
Sex		
Male	67	54.9%
Female	55	45.1%
Age group		
≤60	46	37.7%
>60	76	62.3%
Smoking status		
Current	51	41.8%
Never	71	58.2%
Alcohol use		
Positive	37	30.3%
Negative	85	69.7%
Chewing betel nuts		
Current	2	1.6%
Never	120	98.4%
Disease focus		
Lower gingiva	45	36.9%
Upper gingiva	22	18.0%
Buccal mucosa	55	45.1%
Bone invasion		
Maxilla	23	18.9%
Mandible	42	34.4%
Both	11	9.0%
None	46	37.7%
Skin invasion		
No	115	94.3%
Yes	7	5.7%
Growth pattern of tumor		
Exogenous	29	23.8%
Ulceration type	49	40.2%
Infiltrating type	44	36.1%
Clinical T distribution		
T1	6	4.9%
T2	32	26.2%
T3	8	6.6%
T4a	56	45.9%
T4b	20	16.4%
Clinical N distribution		
N0	79	64.8%
N1	25	20.5%
N2a	6	4.9%
N2b	11	9.0%
N2c	1	0.8%
N3	0	0.0%
Clinical stage		
I	3	2.5%
II	25	20.5%
III	10	8.2%
IV	84	68.8%
Pathological T distribution		
T1	4	3.3%
T2	30	24.6%
T3	12	9.8%
T4a	55	45.1%
T4b	21	17.2%
Tumor invasion depth		
≤5 mm	34	27.9%
5-10 mm	46	37.7%
>10 mm	42	34.4.%
Pathological N distribution		
N0	71	58.2%
N1	16	13.1%
N2a	3	2.5%
N2b	22	18.0%
N2c	0	0.0%
N3a	2	1.6%
N3b	8	6.6%
Histopathological differentiation		
Well	21	17.2%
Moderate	95	77.9%
Poor	6	4.9%
Perineural invasion		
Absence	118	96.7%
Presence	4	3.3%
Extracapsular invasion		
Absence	109	89.3%
Presence	13	10.7%
Titanium plate implantation		
No	79	64.8%
Yes	43	35.2%
Adjuvant radiotherapy		
No	68	55.7%
Yes	54	44.3%
Concomitant chemoradiotherapy		
No	112	91.8%
Yes	10	8.2%

### Tumor and pathological factors

The most common central site of the primary tumor was the buccal mucosa (45.1%). The most common growth patterns of tumors was ulceration type (40.2%). The tumor invaded the bone in 76 (62.3%) patients, the mandible in 42 (34.4%) patients, the maxilla in 23 (18.9%) patients, and the maxilla and the mandible simultaneously in 11 (9.0%) patients. The tumor invaded the skin in 7 (5.7%) patients. Among the clinical distributions, most cases were stage IV (68.8%). Among the pathological T distributions, most cases were pT4a (45.1%). Among the pathological N distributions, most cases were pN0 (58.2%). The most common depth of invasion was 5-10 mm, accounting for 37.7%. The most common histopathological differentiation was moderate differentiated SCC. Extracapsular invasion was found in 13 (10.7%) patients (as shown in Table 1).

### Treatment

All patients underwent surgical treatment with or without adjuvant treatment. Sixty-seven (54.9%) patients underwent supra-omohyoid neck dissection, and 28 (23.0%) underwent modified neck dissection. Only 5 (4.1%) patients required bilateral neck dissection. Another 22 (18.0%) patients did not undergo neck dissection because of early pT stage and no suspicious metastasis lymph nodes in the neck were found in the preoperative clinical examination and imaging data. Sixty-seven (54.9%) patients used flaps for repair and reconstruction because of the large amount of soft tissue removed during surgery, while 30 (24.6%) patients who underwent mandibular segmental resection used bone tissue. The forearm flap (29.5%) was the most common reconstructive technique used during this study period. In 43 (35.2%) patients, titanium plates were implanted during surgery to fix the split mandible or to reconstruct the shape and function of the mandible. Fifty-four patients (44.3%) underwent adjuvant radiotherapy. Only 10 patients (8.2%) underwent postoperative concomitant chemoradiotherapy, while, three patients with positive extracapsular invasion did not undergo concomitant chemoradiotherapy because of patient refusal or poor general condition. The detailed data are summarized in Table 1.

### Survival analysis

A total of 122 patients were included in the survival analysis; 78 (63.9%) patients survived, and 44 (36.1%) patients died. A total of 54 (44.3%) patients experienced relapse within two years after surgery. Regarding the site of relapse, 17 patients (13.9%) had local recurrence, 8 (6.6%) had regional recurrence, 5 (4.1%) had second primary malignancy and 8 (6.6%) had distant metastasis. Because some patients had several events at the same time, 4 patients (3.3%) had loco-regional recurrence, 4 (3.3%) had local recurrence with distant metastasis, and 7 (5.7%) had regional recurrence with distant metastasis. Only 1 patient (0.8%) had loco-regional recurrence and distant metastasis. Surprisingly, the outcome of all patients who underwent regional recurrence was death. Three (2.4%) patients died within 2 year postoperatively due to non cancer-related cause. Overall, the 2-year disease-free survival rate for this cohort was 53.3%.

Of the patients with relapse, 41 patients died of severe illness that could not be treated, and the mortality rate after relapse was 75.9%. The median survival time of patients who died after relapse was 5.0 months (average time: 10.5 months).

Strikingly, 37 (68.5%) patients relapse occurred within one year after surgery. The median time to relapse was 9.0 months (average time: 14.7 months).

There were 23 patients with tumors involving the maxilla; among them, 14 (60.9%) patients developed recurrence, and 12 (52.2%) patients died. There were 42 patients with tumors involving the mandible, of whom 23 (54.8%) experienced recurrence, and 18 (42.9%) died. Tumors invading both the maxilla and mandible simultaneously were found in 11 patients. Seven (63.6%) patients experienced recurrence, and 6 (54.5%) died. The detailed data are summarized in Table 2.

**Table 2:** Prognosis of patients with bone involvement

Category	Recurrence	Death	Total
Bone invasion			76
Maxilla	14(60.9%)	12(52.2%)	23
Mandible	23(54.8%)	18(42.9%)	42
Maxilla and mandible	7(63.6%)	6(54.5%)	11

Of 122 patients, skin invasion was found in 7 patients, 5 patients (71.4%) experienced recurrence, 5 (71.4%) patients died. The average survival time of patients who died was only 19.2 months.

Through the Cox proportional hazards model, it was found that pN stage and bone invasion were significant independent predictors of DFS. The detailed data are summarized in Table 3.

**Table 3:** Univariate and multivariate analyses of prognostic factors influencing DFS

Variable	Hazard ratio	95% Confidence interval	p
Univariate analysis			
Age (≤60 vs. >60)	1.223	0.699-2.139	0.480
Sex (Male vs. Female)	0.580	0.331-1.015	0.057
BMI (<24 vs. ≥24)	0.888	0.507-1.554	0.678
Tobacco use (No vs. Yes)	1.218	0.714-2.080	0.469
Alcohol use (No vs. Yes)	1.394	0.802-2.423	0.239
Diabetes (No vs. Yes)	1.229	0.668-2.260	0.507
Disease focus			
Inferior gingiva	Ref		0.483
Buccal mucosa	0.948	0.515-1.745	
Upper gingiva	1.437	0.697-2.962	
Growth pattern			
Exogenous	Ref		0.242
Ulceration	1.460	0.721-2.957	
Infiltrating	0.884	0.411-1.898	
Bone invasion			
None	Ref		0.005
Maxilla	3.955	1.751-8.936	
Mandible	3.135	1.487-6.613	
Both	3.577	1.342-9.533	
Skin invasion (No vs. Yes)	1.959	0.770-4.986	0.158
Clinical stage			
I	Ref		0.082
II	0.779	0.094-6.474	
III	0.000	0.000	
IV	2.299	0.317-16.686	
pT stage			
pT1	Ref		0.006
pT2	1.115	0.137-9.074	
pT3	0.656	0.059-7.272	
pT4	3.326	0.457-24.190	
pN stage			
pN0	Ref		0.001
pN1	1.108	0.480-2.556	
pN2	1.933	1.008-3.708	
pN3	5.860	2.470-13.903	
Histopathological differentiation			
Well	Ref		0.591
Moderate	0.614	0.163-2.318	
Poor	0.899	0.278-2.902	
Tumor invasion depth			
≤5 mm	Ref		0.111
5-10 mm	1.599	0.765-3.342	
>10 mm	2.159	1.049-4.443	
Perineural invasion (Absence vs. Presence)	2.129	0.663-6.837	0.204
Extracapsular invasion (Absence vs. Presence)	3.069	1.425-6.609	0.004
Postoperative radiotherapy (No vs. Yes)	1.695	0.990-2.901	0.055
Multivariate analysis			
pN stage			
pN0	Ref		0.002
pN1	1.161	0.493-2.736	0.732
pN2	1.844	0.914-3.719	0.087
pN3	5.625	2.299-13.765	0.000
Bone invasion			
None	Ref		0.007
Maxilla	4.185	1.835-9.544	0.001
Mandible	2.770	1.298-5.911	0.008
Both	2.699	0.981-7.429	0.055

### Complications/sequalae

Seventy-seven (63.1%) of 122 patients had postoperative complications/sequalae, and a total of 164 complications/sequalae were found. The most common of these was facial deformities (53 patients, 68.8%). Limitation of mouth opening was found in 32 (41.6%) patients. Thirty (39.0%) patients had nerve injury, of which 19 had numbness of lower lip due to injury to the inferior alveolar nerve, 13 had difficulty lifting the shoulder due to injury to the accessory nerve, and 6 had distortion of commissure due to injury to the marginal mandibular branch of facial nerve. Of these patients, 8 patients had more than one nerve injury. Fifteen (19.5%) patients had postoperative chronic pain. Both flap necrosis and oronasal fistula were found in 3 (3.9%) patients.

A total of 54 patients underwent postoperative radiotherapy, of whom 10 (18.5%) had postoperative radiotherapy complications/sequalae, including oligoptyalism, frequent oral ulcers, infections, skin ulcerations, delayed wound healing and radionecrosis of the jaws.

Of the 43 patients implanted with titanium plates, 18 patients (41.9%) had hardware-related complications/sequalae. Six (33.3%) patients had abnormal sensation, including feeling that the titanium plate was too heavy, felt cold in winter, and felt uncomfortable with the shape of the titanium plate. Eleven (61.1%) patients had exposed titanium plates (1 case of fracture of the titanium plate). And 1 (5.6%) patient had screw loosening. Twenty of 43 patients underwent postoperative radiotherapy, and 7 (35.0%) of them had titanium plate exposure after radiotherapy. Of the other 23 patients who did not undergo radiotherapy, only 4 (17.4%) patients had titanium plate exposure. The data on complications/sequalae are summarized in Table 4.

**Table 4:** Proportion of related complications/sequalae

Category	Number	Proportion
Nerve injury	30	39.0%
Limitation of mouth opening	32	41.6%
Flap necrosis	3	3.9%
Oronasal fistula	3	3.9%
Facial deformity	53	68.8%
Chronic pain	15	19.5%
Post-radiotherapy complications	10 (total: 54)	18.5%
Hardware-related complications	18 (total: 43)	41.9%

## Discussion

Some clinicians and researchers have been exploring the factors affecting the prognosis and quality of life of patients with SCC of the GBC, a subsite of oral cancer with a poor prognosis and a high incidence of postoperative complications/sequalae [14]. However, there are still many controversies. Therefore, in this study, we analyzed 122 SCC patients with GBC to assess its possible influencing factors and try to determine the underlying mechanisms. It is hoped that this study can provide surgeons with useful assistance in future diagnosis and treatment. The characteristics of GBC cancer are summarized in Table 5.

**Table 5:** Characteristics of GBC cancer

Category	Characteristic
Epidemiological	High incidence in Southeast Asia, South-central China，Africa (high consumption area of betel nut)
Clinical manifestation	(1) Large range of lesions (2) Patients with a history of mucosal disease: the depth of tumor invasion is shallow; patients with a history of betel nut chewing: the depth of lesion invasion is deeper
Surgical features	(1) The concept of bone resection is consistent with gingival cancer (2) The concept of the buccal resection is the same as that of buccal cancer (3) The marginal mandibular branch of facial nerve is not considered to preserve for GBC cancer involving the mandible
Prognosis	High recurrence and metastasis rate, poor prognosis
Complication	Due to the insufficient thickness of the soft tissue covering the buccal, post-radiotherapy complications and hardware related complications are high, especially abnormal sensation and titanium plates exposure

We believe that the reasons for the poor prognosis and high complication/sequalae rates are that, on the one hand, the majority of these patients are in the advanced stage, and the progression of the disease leads to poor prognosis; on the other hand, the tumor involves multiple sites, which increases the difficulty of the operation. In previous studies, we found that cancer involving the buccal mucosa was more aggressive, as patients with buccal cancer had a worse prognosis than those with other oral cancers [4]. In addition, in some patients with rigid internal fixation (RIF), the implantation of titanium plates and adjuvant radiotherapy might increase the incidence of postoperative complications [15]. However, there are still many factors that deserve attention.

**Fig. 2:**
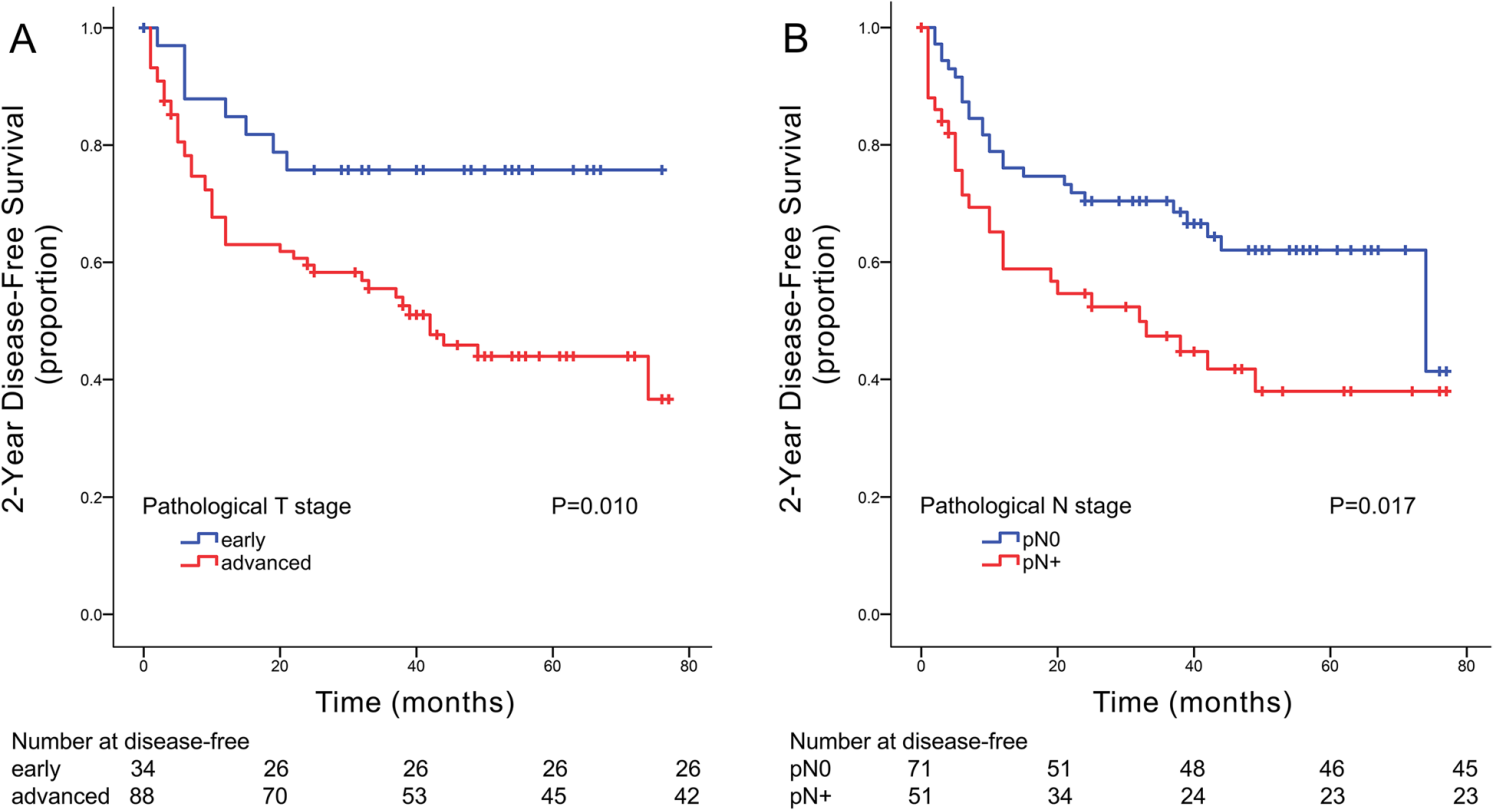
K-M curves drawn by dividing pathological T stage into early (pT1 and pT2) and late (pT3 and pT4) stages (**A**); K-M curves drawn by dividing pathological N stage into pN0 and pN+ (pN1, pN2 and pN3) (**B**)

Guerra et al. [16] reported that the prognosis of early-stage oral cancer was much better than that of advanced-stage oral cancer. In this study, the 2-year DFS rate of early-stage patients was much higher than that of advanced-stage patients (Fig. 2).

Scholars [5] generally considered that patients with poor histopathological differentiation had the worst prognosis, and there was much evidence regarding the importance of tumor histopathological differentiation in predicting recurrence in some studies [17–19]. However, univariate Cox risk regression model analysis in our research resulted that tumor histopathological grade was not an independent predictor of DFS, and other studies did not provide any evidence [20].

Fang et al. [21] found that tumor invasion to the skin was a significant independent predictor in the Cox proportional hazards model. Upon microscopic examination, most thick tumors exhibit direct penetration from the mucosal layers to the cutaneous layers. Radical surgery with adequate margins is difficult to obtain in these patients because the infiltrative tumors might have spread along the subdermal lymphatics and left microscopic residual disease in the postoperative buccal flaps or skin layer. Therefore, they propose that patients with tumors involving the skin should start adjuvant radiotherapy as early as possible. However, in this study, tumor invasion to the skin was not a significant independent predictor, possibly because the sample size of this study was not large enough.

**Fig. 3:**
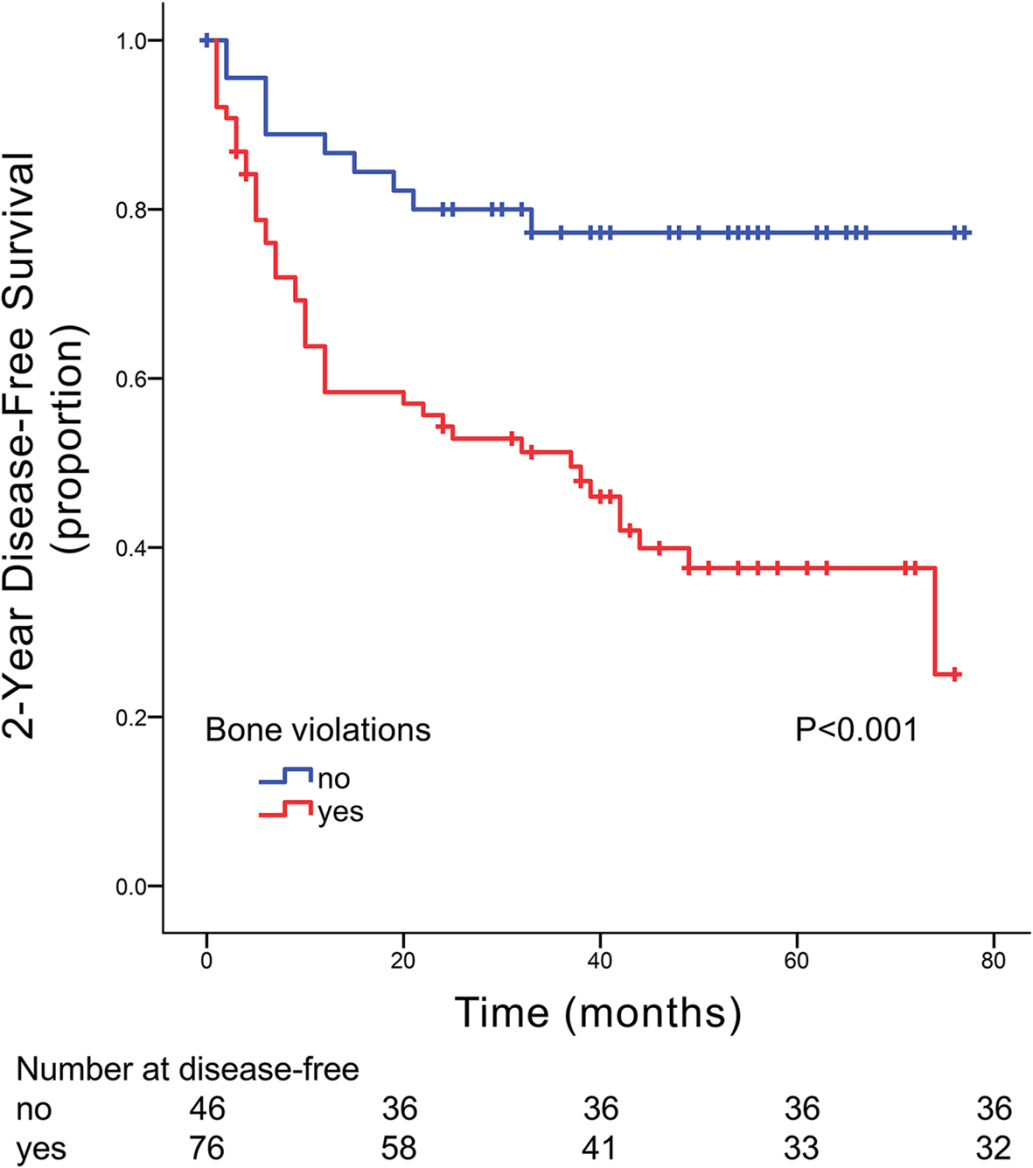
K-M curves comparing whether the jaw was involved

Many studies have found that maxillary gingival SCCs exhibit a higher loco-regional failure rate than mandibular gingival SCCs [22–24]. The research by Feng et al. [25] also confirmed that middle-stage and advanced-stage SCCs of the maxillary gingiva have more aggressive behavior than oral cancers of other locations. In multivariate analysis of this study, bone invasion was found to be an independent prognostic factor for DFS. We found that the prognosis of patients with maxillary invasion was significantly worse than that of patients with mandibular invasion (Fig. 3). This may be because of the thin layer of soft tissue covering the maxilla and because tumor cell invasion of the adjacent bone can occur faster [26]. Soluble factors released by various cell types in tumors to the extracellular milieu through the paracrine signaling function to induce tumor invasive growth [27]. The blood circulation of the maxilla is abundant, tumor cells and soluble factors easily metastasize through blood vessels [25]. It was difficult to achieve en bloc resection and clean margins [28] because tumor cells invaded the submucosa, extending into the pterygoid process and the base of the skull. Furthermore, some patients chose relatively conservative surgical resection because of aesthetics, function, and reconstruction problems. In addition, Yang et al. [26] found that patients who underwent conservative surgical treatment had a poor prognosis. Umeda et al. [29] considered that there were two lymph node drainage routes from the maxilla to the neck. One derived from the maxillary gingiva crosses through the buccal nodes and finally into the submandibular nodes. The other, derived from the soft palate or nasal floor, crosses through the parapharyngeal or retropharyngeal space and finally into the upper jugular nodes. There may be residual tumor cells in the lymph fluid. However, it is difficult to determine whether this phenomenon is a result of lymphatic flow or other specific anatomic structures, and more research is needed in the future for confirmation.

In addition to some common postoperative complications/sequalae, such as facial deformities, nerve injury, limitation of mouth opening, and difficulties in shoulder lifting, we mainly discuss some special complications/sequalae. The loco-regional control rates of OSCC treated with adjuvant radiotherapy range from 42% to 74% [21]. However, radiotherapy can also cause damage to the skin, bones, oral mucosa, and salivary glands and subsequently cause oligoptyalism, frequent oral ulcers, infections, skin ulcerations, delayed wound healing and radionecrosis. In this study, almost 20.0% of patients receiving postoperative radiotherapy had post-radiotherapy complications. Nevertheless, adjuvant radiotherapy still plays an important role in improving the prognosis and decreasing the recurrence rate of GBC cancer [26].

Scholars generally considered that at least 10 mm postoperative mandibular bone height needed to be preserved after mandibular marginal resection to prevent mandibular fracture [30]. Okuyama et al. [31] further found that the preserved mandibular bone should be reinforced with hardware or bone transplantation in patients with mandibular body height preservation ratio of less than 0.3, more than 20 remaining teeth after surgery, and intraoral inferior alveolar canal exposure. Some studies have shown that hardware-related complications/sequalae might occur in up to 45% of cases and include hardware exposure (4–46%), loose screws (0.8–5.8%), and hardware fractures (0–3.3%) [32]. In this study, about 40.0% of the patients who had titanium plates implanted had hardware-related complications/sequalae. Patients receiving postoperative radiotherapy were more likely to have hardware-related complications/sequalae than patients not receiving radiotherapy in this study. This may be caused by the insufficient thickness of the soft tissue on the surface of the hardware [33]. Postoperative radiotherapy will further aggravate skin and cause subcutaneous tissue atrophy and fibrosis [34]. Ryu et al. [35] also confirmed through simulated radiotherapy experiments that the highest radiation dose occurs at the junction of the hardware and the tissue. The wound on the surface of the hardware cannot heal normally, the skin becomes necrotic, and the hardware is eventually exposed.

Chronic pain is also one of the long-term complications that is often overlooked. The probability of chronic pain after surgery for OSCC patients is approximately 30% to 60.38% [36]. This may be because the nerve is damaged by the operation [37]. The scars formed after the operation cause deep muscles and nerves to adhere, causing the nerves to be continuously excited, thus causing pain [38]. Moreover, local inflammation caused by IL-1, IL-6 and TGF-β induced by postoperative radiotherapy destroys nociceptors [39].

This study comprehensively analyzed the basic information, prognosis and complications/sequalae of SCC in GBC patients in our institution. However, there are also some shortcomings, such as the bias caused by the small sample size and the short follow-up time, and no postoperative quality of life indicators and body image indicators of patients. In the future, as the sample size expands and the follow-up time increases, we will continue to conduct in-depth research on this issue. We are also actively researching a method that can reduce radiotherapy complications and hardware-related complications and alleviate postoperative chronic pain.

## Conclusions

SCC of GBC has a high recurrence rate, high complication/sequalae rate and poor prognosis. Pathological N stage and bone invasion were significant independent prognostic predictors of DFS in this study. Patients with maxillary bone involvement had a higher loco-regional failure rate and a worse prognosis than those with mandibular bone involvement. Postoperative radiotherapy patients were more prone to long-term complications/sequalae such as hardware-related complications/sequalae and chronic pain.

## Data Availability

The datasets used and/or analyzed during the current study are available from the corresponding author on reasonable request.

## Abbreviations

OSCC: Oral squamous cell carcinoma
GBC: Gingivobuccal complex
SCC: Squamous cell carcinoma
POROMS: Prospective, Observational, Real-world Oral Malignant Tumors Study
AJCC: American Joint Committee on Cancer
NCCN: National Comprehensive Cancer Network
DFS: Disease-free survival
K-M: Kaplan-Meier
BMI: Body mass index
RIF: Rigid internal fixation
IL: Interleukin
TGF: Transforming growth factor
